# Interventions for treatment of COVID-19: a protocol for a living systematic review with network meta-analysis including individual patient data (The LIVING Project)

**DOI:** 10.1186/s13643-020-01371-0

**Published:** 2020-05-09

**Authors:** Sophie Juul, Niklas Nielsen, Peter Bentzer, Areti Angeliki Veroniki, Lehana Thabane, Adam Linder, Sarah Klingenberg, Christian Gluud, Janus Christian Jakobsen

**Affiliations:** 1 Copenhagen Trial Unit – Centre for Clinical Intervention Research, Rigshospitalet, Copenhagen University Hospital, Blegdamsvej 9, 2100 Copenhagen Ø, Denmark; 2 Department of Clinical Sciences Lund, Anesthesia & Intensive care, Helsingborg Hospital, Lund University, Lund, Sweden; 3 Department of Primary Education, School of Education, University of Ioannina, Ioannina, Greece; 4 Knowledge Translation Program, Li Ka Shing Knowledge Institute, St. Michael’s Hospital, Toronto, Ontario Canada; 5 Department of Health Research Methods, Evidence, and Impact, McMaster University, Hamilton, ON Canada; 6 Faculty of Health Sciences, University of Southern Denmark, Odense, Denmark

## Abstract

**Background:**

COVID-19 is a rapidly spreading virus infection that has quickly caused extensive burden to individual, families, countries, and the globe. No intervention has yet been proven effective for the treatment of COVID-19. Some randomized clinical trials assessing the effects of different drugs have been published, and more are currently underway. There is an urgent need for a living, dynamic systematic review that continuously evaluates the beneficial and harmful effects of all available interventions for COVID-19.

**Methods/design:**

We will conduct a living systematic review based on searches of major medical databases (e.g., MEDLINE, EMBASE, CENTRAL) and clinical trial registries from their inception onwards to identify relevant randomized clinical trials. We will update the literature search once a week to continuously assess if new evidence is available. Two review authors will independently extract data and perform risk of bias assessment. We will include randomized clinical trials comparing any intervention for the treatment of COVID-19 (e.g., pharmacological interventions, fluid therapy, invasive or noninvasive ventilation, or similar interventions) with any comparator (e.g., an “active” comparator, standard care, placebo, no intervention, or “active placebo”) for participants in all age groups with a diagnosis of COVID-19. Primary outcomes will be all-cause mortality and serious adverse events. Secondary outcomes will be admission to intensive care, mechanical ventilation, renal replacement therapy, quality of life, and non-serious adverse events. The living systematic review will include aggregate data meta-analyses, Trial Sequential Analyses, network meta-analysis, and individual patient data meta-analyses. Risk of bias will be assessed with domains, an eight-step procedure will be used to assess if the thresholds for clinical significance are crossed, and the certainty of the evidence will be assessed by Grading of Recommendations, Assessment, Development and Evaluations (GRADE).

**Discussion:**

COVID-19 has become a pandemic with substantial mortality. A living systematic review evaluating the beneficial and harmful effects of pharmacological and other interventions is urgently needed. This review will continuously inform best practice in treatment and clinical research of this highly prevalent disease.

**Systematic review registration:**

PROSPERO CRD42020178787

## Background

### Description of participants

In early December 2019, a novel coronavirus named severe acute respiratory syndrome coronavirus 2 (SARS-CoV-2) caused an international outbreak of the respiratory illness COVID-19 [1]. Since the initial outbreak in China, SARS-CoV-2 has spread globally, and COVID-19 has now been labeled a public health emergency of international concern by the World Health Organization [2]. The full spectrum of COVID-19 ranges from subclinical infection over mild, self-limiting respiratory tract illness to severe progressive pneumonia, multiorgan failure, and death [3]. Severe disease onset might result in death due to massive alveolar damage and progressive respiratory failure [4–6]. Currently, COVID-19 is spreading rapidly through Europe and North America [7]. As of April 17, 2020, there were 2,074,529 confirmed patients, 139,378 confirmed deaths, and 213 countries, areas, or territories with COVID-19 according to the World Health Organization [8].

### Description of interventions

There is currently no proven treatment for COVID-19 [7]. To control the growing COVID-19 pandemic, we rely on quarantine, isolation, and infection-control measures to prevent disease spread [7], and on supportive care including oxygen and mechanical ventilation for infected patients.

Today, different drugs exist that are currently being assessed for patients with COVID-19: remdesivir (used to treat Ebola virus disease and Marburg virus infections), drugs containing lopinavir and ritonavir (used to treat HIV/AIDS), chloroquine phosphate or hydroxychloroquine (used to treat malaria), tocilizumab (used to treat rheumatoid arthritis), corticosteroids, stem cells, and other types of interventions [9]. More examples of potential interventions for treatment of COVID-19 can be found in Table 1.

**Table 1:** Examples of potential interventions for treatment of COVID-19

• Anti-infectious agents including antiviral treatments such as remdesivir, lopinavir, ritonavir, oseltamivir, favipiravir, umifenovir, chloroquine, hydroxychloroquine, and azithromycin. • Immunomodulators such as interferon alpha, interferon beta, nivolumab, and tocilizumab. • Non-specific immunomodulators such as corticosteroids, polyclonal antibodies, convalescent plasma, and colchicine. • Supportive treatments for patients admitted to intensive care, such as high-flow nasal canula, non-invasive ventilation, protective mechanical ventilation, and extra corporal membrane oxygenation (ECMO). • General interventions for viral infection such as vitamin C, zinc, and selenium.	

Randomized clinical trials assessing the effects of interventions for COVID-19 are urgently needed. Several randomized clinical trials are underway. According to an online global COVID-19 clinical trial tracker available at www.covid19-trials.org, there are currently 590 trials registered worldwide.

However, a single trial can rarely validly assess the effects of any intervention, and there is an urgent need to continuously surveil the literature and update the aggregated evidence base so that effective interventions, if such exist, are implemented clinically [10]. We have searched in published protocols, PROSPERO, and relevant websites, and we have identified more than 400 ongoing projects aiming at synthesizing COVID-19 evidence. It is currently not possible to assess the methodological quality of all these projects because most of these are only summaries of methodologies and not detailed published protocols. Furthermore, multiple new projects are launched every day. We identified three important reviews that are comparable to our present project. The first is a literature review published in JAMA using PubMed to identify relevant English-language articles published through March 25, 2020, on pharmacological interventions for COVID-19. The search resulted in 1315 total articles. Due to the lack of randomized clinical trials, the authors also included case reports, case series, and review articles. However, this review was only a narrative review without meta-analytic methods and Trial Sequential Analysis [11].

The second project is a living mapping of ongoing randomized clinical trials with network meta-analysis on all interventions for COVID-19. This project was registered in Zenodo on April 8, 2020, (https://zenodo.org/record/3744600#.Xp8U0MgzZPY), and the authors are producing and disseminating preliminary results through an open platform (https://covid-nma.com/). However, this review includes both prevention and treatment and does not use Trial Sequential Analysis or similar methods to handle problems with multiplicity (repeating updating of meta-analysis, multiple comparisons due to inclusion of multiple interventions, assessing multiple outcomes).

The third project is a preprint of a rapid review assessing the effectiveness and safety of antiviral antibody treatments for COVID-19 published in medrXiv [12]. Fifty-four studies were included in the review: three controlled trials, 10 cohort studies, seven retrospective medical record/database studies, and 34 case reports or series. These studies included patients with severe acute respiratory syndrome (SARS, *n* = 33), Middle East respiratory syndrome (MERS, *n* = 16), COVID-19 (*n* = 3), and unspecified coronavirus (*n* = 2). The most common treatment was ribavirin (*n* = 41), followed by oseltamivir (*n* = 10), and the combination of lopinavir/ritonavir (*n* = 7). The authors conclude that current evidence for the effectiveness and safety of antiviral therapies for coronavirus is inconclusive and suffers from a lack of well-designed prospective trials. However, this review was only a narrative review without meta-analytic methods and Trial Sequential Analysis.

None of the numerous identified previous registered protocols includes individual patient data in a living systematic review format taking into account both risks of random errors “play of chance” and risks of systematic errors “bias” [13, 14].

A living systematic review allows us to incorporate relevant new evidence as it becomes available, thereby decreasing the timespan from evidence to clinical practice, which is crucial in this international health crisis. The present living systematic review with aggregate meta-analyses, Trial Sequential Analyses, network meta-analysis, and individual patient data meta-analyses aims at continuously forming the basis for evidence-based guideline recommendations for the treatment of COVID-19, taking bias risk (systematic errors), play of chance (random errors), and certainty of the findings into consideration.

## Methods

The protocol is reported in accordance with the reporting guideline provided in the Preferred Reporting Items for Systematic Reviews and Meta-Analysis Protocols (PRISMA-P) statement (see Additional file 1**)** [15, 16], and is registered in the PROSPERO database (CRD42020178787). The review will be carried out following recommendations outlined in The Cochrane Handbook of Systematic Review of Interventions [14].

### Criteria for considering studies for this review

#### Types of studies

We will include randomized clinical trials, irrespective of publication status, publication year, and language. We will not include quasi-randomized trials and observational studies.

#### Types of participants

Participants in all age groups with a diagnosis of COVID-19 and the associated SARS-CoV-2 virus as confirmed by laboratory tests (such as r*everse transcription polymerase chain reaction* (RT-PCR)) will be eligible. Participants will be included irrespective of sex and comorbidities.

#### Types of interventions

##### Experimental group

We will include any intervention used to try to treat COVID-19, i.e., all interventions listed in Table 1 or any other intervention irrespective of dose and duration of administration. We will not include traditional Chinese medicines or preventive interventions (vaccination etc.) as these interventions deserve their own assessments in similar protocols to the present.

##### Control group

We will include randomized clinical trials with any control group, i.e., head-to-head comparisons (any “active” comparator), placebo, “active placebo” (a matching placebo that produces noticeable adverse effects that may convince the participant being treated and blinded outcome assessors that the participants are receiving an active intervention), usual care (or similar terms), or no intervention. We will accept any of these control interventions irrespective of dose and duration of administration.

##### Cointervention

We will accept any cointervention, if the cointervention is intended to be delivered similarly in the intervention and control groups.

### Outcome measures

#### Primary outcomes

1. All-cause mortality.
2. Proportion of participants with one or more serious adverse events. We will use the International Conference on Harmonization of technical requirements for registration of pharmaceuticals for human use—Good Clinical Practice (ICH-GCP) definition of a serious adverse event, which is any untoward medical occurrence that resulted in death, was life-threatening, required hospitalization or prolonging of existing hospitalization, and resulted in persistent or significant disability or jeopardized the participant [17]. If the trialists do not use the ICH-GCP definition, we will include the data if the trialists use the term “serious adverse event.” If the trialists do not use the ICH-GCP definition nor use the term serious adverse event, then we will also include the data, if the event clearly fulfills the ICH-GCP definition for a serious adverse event. We will exploratorily assess each type of serious adverse event separately.

#### Secondary outcomes

1. Proportion of participants admitted to intensive care (as defined by trialists).
2. Proportion of participants receiving mechanical ventilation (as defined by trialists).
3. Proportion of participants receiving renal replacement therapy (as defined by trialists).
4. Quality of life assessed on any valid continuous scale.
5. Proportion of participants with one or more non-serious adverse events (any adverse event not classified as serious). We will exploratorily assess each adverse event separately.

#### Assessment time points

We will assess all outcomes at maximum follow-up.

### Search methods for identification of studies

#### Electronic searches

An experienced librarian will search Cochrane Central Register of Controlled Trials (CENTRAL), Medical Literature Analysis and Retrieval System Online (MEDLINE), Excerpta Medica database (EMBASE), Latin American and Caribbean Health Sciences Literature (LILACS), Science Citation Index Expanded (SCI-EXPANDED), Conference Proceedings Citation Index—Science (CPCI-S), Chinese Biomedical Literature Database (CBM), China Network Knowledge Information (CNKI), Chinese Science Journal Database (VIP), and Wafang Database to identify relevant trials. We will search all databases from their inception to the present. Trials will be included irrespective of language, publication status, publication year, and publication type. For a detailed search strategy for all electronic searches, see Additional file 2.

#### Searching other resources

The reference lists of relevant trial publications will be checked for any unidentified randomized clinical trials. We will contact the authors of included trials by email asking for unpublished randomized clinical trials. To identify unpublished trials, we will search clinical trial registries (e.g., clinicaltrials.gov, clinicaltrialregister.eu, who.int/ictrp, chictr.org.cn) of Europe, the USA, and China, and websites of pharmaceutical companies, websites of US Food and Drug Administration (FDA), and European Medicines Agency (EMA), and we will request FDA, EMA, and national medicines agencies to provide all publicly releasable information about relevant randomized clinical trials of COVID-19 medication that were submitted for marketing approval. We will also search the COVID-19 Study Registry (https://covid-19.cochrane.org/) and COVID-evidence (https://covid-evidence.org/).

Additionally, we will hand search conference abstracts from COVID-19 conferences for relevant trials. We will also include unpublished and gray literature trials if we identify these and assess relevant retraction statements and errata for included trials. We will search preprint servers (bioRxiv, medRxiv) for unpublished trials.

We will contact all trial authors to receive individual patient data so individual patient data meta-analysis is possible. Given the urgency, we probably have more chances to get individual patient data than normally. We will also contact individual patient data databases, such as the Yale University Open Data Access (YODA) and Clinical Study Data Request (CSDR), to increase our chances to obtain relevant data. We will seek to obtain individual patient data regarding all our outcomes and all other adjustment variables used in the analyses of each trial.

#### Living systematic review

**Fig. 1:**
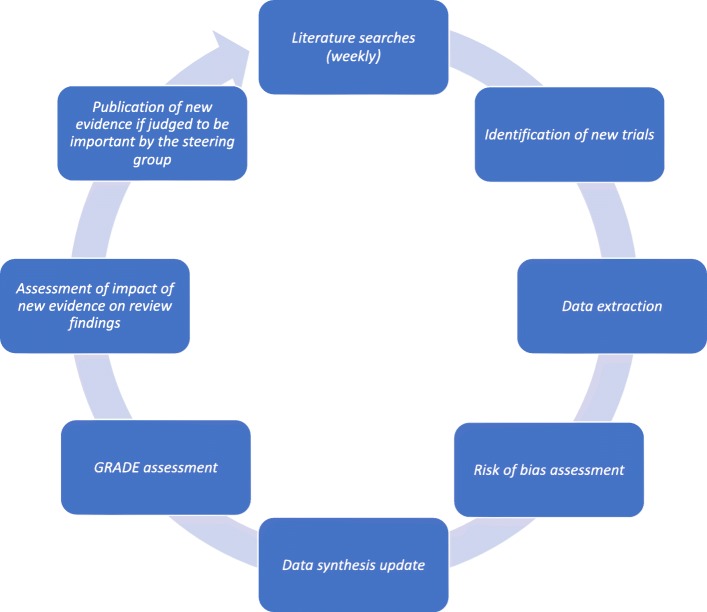
Living systematic review work flow

A living systematic review is defined as a systematic review which is continually updated and incorporates relevant new evidence as it becomes available [18]. This methodology may be particularly important in the COVID-19 pandemic, where research evidence is emerging rapidly, current evidence is uncertain, and new research may change policy or practice decisions [18]. There are four fundamental differences between conventional systematic reviews and living systematic reviews: publication format, work processes, author team management, and statistical methods [19]. In this living systematic review, two independent investigators will receive an updated literature search file and include relevant newly published or unpublished trials once a week. The relevant meta-analyses, Trial Sequential Analyses, and network meta-analysis will continuously be updated, and if new evidence is available (judged by the steering committee), the results will be published. Every month, the steering committee will discuss whether searching once a week is necessary. The living systematic review process will be initiated April 27, 2020. For an illustration of the living systematic review work flow, see Fig. 1.

### Data collection

We will perform and report the review as recommended by the Preferred Reporting Items for Systematic Reviews and Meta-Analyses (PRISMA) statement [20]. Analyses will be performed using Stata version 16 (StataCorp LLC, College Station, TX, USA) [21] and trial sequential analysis [22, 23].

#### Selection of randomized clinical trials

Two review authors will independently screen titles and abstracts. We will retrieve all relevant full-text study reports/publications, where two review authors will independently screen the full-texts and will record reasons for exclusion of the ineligible studies. The same two review authors will resolve any disagreements through discussion, or if required, they will consult a third person (JCJ).

#### Data extraction and management

Two review authors will independently extract data from included trials in a predefined form. Disagreements will be resolved by discussion with a third author (JCJ). The two review authors will assess duplicate publications and companion papers of a trial together to evaluate all available data simultaneously (maximize data extraction, correct bias assessment). Each trial will be named after the first author and year of the primary publication, and all secondary publications will be classified under that name.

We will contact the trial authors by email to specify any missing data, which may not be reported sufficiently or not at all in the publication.

#### Trial characteristics

We will extract the following data: bias risk components (as defined below), trial design (parallel, factorial, or crossover), number of intervention groups, length of follow-up, estimation of sample size, inclusion and exclusion criteria.

#### Participant characteristics

We will extract the following data: number of randomized participants, number of participants with comorbidities and types of comorbidities, number of analyzed participants, number of participants lost to follow-up/withdrawals/crossover, age range (mean or median), and sex ratio.

#### Experimental intervention characteristics

We will extract the following data: type of experimental intervention, dose of intervention, duration of intervention.

#### Control intervention characteristics

We will extract the following data: type of control intervention, dose of intervention, duration of intervention.

#### Outcomes

All outcomes listed above will be extracted from each randomized clinical trial. For each outcome, we will identify if outcomes are missing, inappropriately measured, or selectively reported according to the criteria described later in the “missing outcome data” bias domain, the “risk of bias in measurement of the outcome” bias domain, and the “risk of bias in selection of the reported result” bias domain.

#### Notes

We will search for information regarding industry funding of either personal or academic activities for each trial author. We will judge a publication at high risk of for-profit bias if a trial is sponsored by the industry or if just one author has affiliation to the industry. We will note in the “Characteristics of included studies” table if outcome data were not reported in a usable way. Two review authors will independently transfer data into the Stata file [21]. Disagreements will be resolved through discussion, or if required, we will consult with a third author (JCJ).

### Assessment of risk of bias in the included studies

Our bias risk assessment will be based on the Cochrane Risk of Bias tool—version 2 (RoB 2) as recommended in The Cochrane Handbook of Systematic Reviews of Interventions [14]. We will evaluate the methodology in respect of the following bias domains:

#### Bias arising from the randomization process

This domain encompasses allocation sequence generation and concealment as well as baseline differences between the trial arms.

##### Low risk of bias

Allocation was adequately concealed, AND there are no baseline imbalances across intervention groups at baseline appear to be compatible with chance, AND an adequate (random or otherwise unpredictable) method was used to generate allocation sequence, OR there is no information about the method used to generate the allocation sequence

##### Some concerns

Allocation was adequately concealed, AND there is a problem with the method of sequence generation, OR baseline imbalances suggest a problem with the randomization process, OR no information is provided about concealment of allocation, AND baseline imbalances across intervention groups appear to be compatible with chance, OR no information to answer any of the signaling questions

##### High risk of bias

Allocation sequence was not concealed, OR no information is provided about concealment of allocation sequence, AND baseline imbalances suggest a problem with the randomization process.

#### Bias due to deviation from intended interventions

##### Low risk of bias

Participants, carers, and personnel were unaware of intervention groups during the trial, OR participants, carers, or personnel were aware of intervention groups during the trial but any deviations from intended intervention reflected usual practice, OR participants, carers, or personnel were aware of intervention groups during the trial but any deviations from intended intervention were unlikely to impact on the outcome, AND no participants were analyzed in the wrong intervention groups (that is, on the basis of intervention actually received rather than of randomized allocation).

##### Some concerns

Participants, carers, or personnel were aware of intervention groups and there is no information on whether there were deviations from usual practice that were likely to impact on the outcome and were imbalanced between intervention groups, OR some participants were analyzed in the wrong intervention groups (on the basis of intervention actually received rather than of randomized allocation) but there was little potential for a substantial impact on the estimated effect of intervention.

##### High risk of bias

Participants, carers, or personnel were aware of intervention groups, and there were deviations from intended interventions that were unbalanced between the intervention groups and likely to have affected the outcome, OR some participants were analyzed in the wrong intervention groups (on the basis of intervention actually received rather than of randomized allocation), and there was potential for a substantial impact on the estimated effect of intervention.

#### Bias due to missing outcome data

##### Low risk of bias

No missing data OR non-differential missing data (similar proportion of and similar reasons for missing data in compared groups) OR evidence of robustness of effect estimate to missing data (based on adequate statistical methods for handling missing data and sensitivity analysis)

##### Some concerns

An unclear degree of missing data or unclear information on proportion and reasons for missingness in compared groups AND there is no evidence that the effect estimate is robust to missing data.

##### High risk of bias

A high degree of missing data AND differential missing data (different proportion of or different reasons for missing data in compared groups) AND there is no evidence that the effect estimate is robust to missing data.

#### Bias in measurement of outcomes

##### Low risk of bias

The outcome assessors were unaware of the intervention received by study participants, OR the outcome assessors were aware of the intervention received by study participants, but the assessment of the outcome was unlikely to be influenced by knowledge of the intervention received.

##### Some concerns

There is no information available to determine whether the assessment of the outcome is likely to be influenced by knowledge of the intervention received.

##### High risk of bias

The assessment of the outcome was likely to be influenced by knowledge of the intervention received by study participants*.*

#### Bias arising from selective reporting of results

##### Low risk of bias

Reported outcome data are unlikely to have been selected, on the basis of the results, from multiple outcome measurements (e.g., scales, definitions, time points) within the outcome domain, and reported outcome data are unlikely to have been selected, on the basis of the results, from multiple analyses of the data.

##### Some concerns

There is insufficient information available to exclude the possibility that reported outcome data were selected, on the basis of the results, from multiple outcome measurements (e.g., scales, definitions, time points) within the outcome domain, or from multiple analyses of the data. Given that analysis intentions are often unavailable or not reported with sufficient detail, we anticipate that this will be the default judgment for most trials.

##### High risk of bias

Reported outcome data are likely to have been selected, on the basis of the results, from multiple outcome measurements (e.g., scales, definitions, time points) within the outcome domain, or from multiple analyses of the data (or both).

#### Overall assessment of risk of bias

##### Low risk of bias

The study is judged to be at low risk of bias for all domains for this result.

##### High risk of bias

The study is judged to be at high risk of bias or to be at some concerns in at least one domain for this result. Our subgroup analysis will compare the intervention effect of trials at low risk of bias with trials at high risk of bias, that is one or more domains at some concern or high risk of bias.

We will assess the domains “missing outcome data,” “risk of bias in measurement of the outcome,” and “risk of bias in selection of the reported result” for each outcome result. Thus, we can assess the bias risk for each outcome assessed in addition to each trial. Our primary conclusions will be based on the results of our primary outcome results with overall low risk of bias. Both our primary and secondary conclusions will be presented in the summary of findings tables.

### Differences between the protocol and the review

We will conduct the review according to this published protocol and report any deviations from it in the “Differences between the protocol and the review” section of the systematic review.

### Measurement of treatment effect

#### Dichotomous outcomes

We will calculate risk ratios (RRs) with 95% confidence interval (CI) for dichotomous outcomes, as well as the trial sequential analysis-adjusted CIs (see below).

#### Continuous outcomes

We will calculate the mean differences (MDs) and consider calculating the standardized mean difference (SMD) with 95% CI for continuous outcomes. We will also calculate trial sequential analysis-adjusted CIs (see below).

### Dealing with missing data

We will use intention-to-treat data if provided by the trialists [24]. We will, as the first option, contact all trial authors to obtain any relevant missing data (i.e., for data extraction and for assessment of risk of bias, as specified above), when individual patient data is not available.

#### Dichotomous outcomes

We will not impute missing values for any outcomes in our primary analysis. In our sensitivity analyses (see paragraph below), we will impute data.

#### Continuous outcomes

We will primarily analyze scores assessed at single time points. We will analyze change from baseline scores using a MD if the same scale is used across studies. For different measurement scales in the same analysis model, we will use the SMD effect size. In case some studies do not report change scores, but provide follow-up values, we will combine them together in a single model using MD [14]. If standard deviations (SDs) are not reported, we will calculate the SDs using relevant trial data (e.g., *P* values), if available. We will not use intention-to-treat data if the original report did not contain such data, per protocol data will then be used. In our best-worst worst-best scenarios (see paragraph below) for continuous outcomes, we will impute data.

### Assessment of heterogeneity

We will primarily investigate forest plots to visually assess any sign of heterogeneity. We will secondly assess the presence of statistical heterogeneity using I^2^ statistic [14, 25, 26] and restricted maximum likelihood method [27, 28]. We will investigate evident heterogeneity through subgroup analyses (see “Subgroup analyses and integration of heterogeneity” section below). We may ultimately decide that a meta-analysis should be avoided if heterogeneity is high [14].

### Assessment of reporting biases

We will use a funnel plot to assess reporting bias if ten or more trials are included [14]. We will visually inspect funnel plots to assess the risk of bias. We are aware of the limitations of a funnel plot (i.e., a funnel plot assesses bias due to small sample size) [14]. From this information, we will assess possible reporting bias. For dichotomous outcomes, we will test asymmetry with the Harbord test [29] if τ^2^ is less than 0.1 and with the Rücker test if τ^2^ is more than 0.1 [14]. For continuous outcomes, we will use the regression asymmetry test [30] and the adjusted rank correlation [31].

### Unit of analysis issues

We will only include randomized clinical trials. For trials using crossover design, only data from the first period will be included [14, 32]. There will therefore not be any unit of analysis issues. We will not include cluster randomized trials.

### Data synthesis

All types of interventions will be included in the network meta-analysis. When analyzing individual patient data meta-analyses, aggregate data meta-analyses, and Trial Sequential Analyses, the results of each type of intervention will be analyzed separately.

We will use intention-to-treat data in all analyses. We will consider using multiple imputation techniques as recommended by Jakobsen et al. [24]. Please consult this publication for a detailed description of the handling of missing data. We will present best-worst and worst-best case scenarios if it is not valid to ignore missing data [13]. Best-worst and worst-best case scenarios assess the potential range of impact of the missing data for the trial results. In the “best-worst” case scenario, it is assumed that all patients lost to follow-up in the intervention group have had a beneficial outcome (have survived, had no poor functional outcome, and so forth), and all those with missing outcomes in the control group have had a harmful outcome (have not survived, have had poor functional outcome, and so forth) [13]. Conversely, in the “worst-best” case scenario, it is assumed that all patients who were lost to follow-up in the experimental group have had a harmful outcome, and that all those lost to follow-up in the control group have had a beneficial outcome [13]. When continuous outcomes are used, a “beneficial outcome” will be defined as the group mean plus two SDs of the group mean, and a “harmful outcome” will be defined as the group mean minus two SDs of the group mean [13].

#### Aggregate data meta-analysis

We will undertake the meta-analyses according to the *Cochrane Handbook of Systematic Reviews of Interventions* [14], Keus et al. [33], and the eight-step assessment suggested by Jakobsen et al. [13]. We will use the statistical software Stata version 16 [21] to analyze data (command: meta). We will assess our intervention effects with both a random-effects meta-analysis [34] and fixed-effect meta-analysis for each treatment comparison separately [35]. We will primarily use the more conservative point estimate of the two [13]. The more conservative point estimate is the estimate with the highest *P* value. We will assess a total of two primary outcomes, and we will therefore consider a *P* value of 0.03 or less as the threshold for statistical significance [13]. We will investigate possible heterogeneity through subgroup analyses. We will use the eight-step procedure to assess if the thresholds for significance are crossed [13]. Where multiple trial arms are reported in a single trial, we will include only the relevant arms. If two comparisons are combined in the same meta-analysis, we will halve the control group to avoid double-counting [14]. Trials with a factorial design will be included. In case of, e.g., a 2 × 2 factorial designed trial, the two groups receiving COVID-19 intervention will be considered experimental groups, while the two groups receiving an “active” comparator, placebo, standard care, “active placebo”, or no intervention will be considered control groups.

#### Trial Sequential Analysis

Due to the continuous inclusion of new trials and hence repetitive testing of accumulating data when updating reviews, there is an increased risk of type I error. We wish to control the risks of both type I errors and type II errors. We will therefore perform Trial Sequential Analysis on all outcomes, in order to calculate the required information size (that is, the number of participants needed in a meta-analysis to detect or reject a certain intervention effect) and the cumulative Z-curve’s breach of relevant trial sequential monitoring boundaries [22, 23, 36–42]. A more detailed description of trial sequential analysis can be found in the trial sequential analysis manual [23] and at http://www.ctu.dk/tsa/. For dichotomous outcomes, we will estimate the required information size based on the observed proportion of patients with an outcome in the control group (the cumulative proportion of patients with an event in the control groups relative to all patients in the control groups), a relative risk reduction or a relative risk increase of 20% or 10%, an alpha of 2% for all our outcomes, a beta of 10%, and the observed diversity as suggested by the trials in the meta-analysis. For continuous outcomes, we will in the Trial Sequential Analysis use the observed standard deviation (SD), a mean difference equal to the observed SD/2, an alpha of 2% for all outcomes, a beta of 10%, and the observed diversity as suggested by the trials in the meta-analysis.

#### Network meta-analysis

We will obtain information about the interventions of interest either from head-to-head trials, or from trials comparing a COVID-19 intervention with placebo, standard care, no intervention, or “active placebo.” Hence, the synthesis comparator set consists of all the interventions listed in the background section as well as placebo, standard care, no intervention, or “active placebo” trials. Each specific intervention (e.g. specific drug (not class) or intubation) will be analyzed separately and will not be clustered.

We will describe the characteristics of the eligible randomized controlled trials and their populations using frequencies and percentages for categorical data, and means and standard deviations for continuous data. Descriptive statistics will be also generated for each treatment comparison describing important clinical and methodological characteristics (e.g., publication year, participant age). Each outcome dataset will be presented in a different network diagram, where the size of the nodes will be proportional to the total number of randomized participants, and the width of each edge will be weighted according to the number of studies comparing the connected treatments. We will additionally plot the edges of each network according to the average risk of bias per treatment comparison, using green for low, yellow for moderate, and red for high risk of bias.

We anticipate that any participant who meets inclusion criteria is, in principle, equally likely to be randomized to any of the interventions in the synthesis comparator set. Network meta-analysis will be performed using Stata16 under frequentist framework [21] (command: mvmeta) [43]. The network meta-analysis synthesizes evidence for the comparative effectiveness of more than two alternative interventions for the same condition [44]. We will only perform network meta-analysis if a connected network of trials can be conducted. If network meta-analysis is performed, we will, before conducting the network meta-analysis, assess two major assumptions; transitivity and consistency. We will then perform the statistical analysis in five steps. First step is to draw a network geometry in order to overview the network relationship. Second step is to assess for the transitivity assumption across treatment comparisons in the network using boxplots, as well as evaluate the assumption of consistency using the design-by-treatment interaction model as a global test [44, 45]. Third step is to make the network forest plot or interval plot in order to illustrate the summary effect size of the comparative effectiveness among interventions. Fourth step is to calculate cumulative rankings for identifying a superiority among interventions. Last step is to evaluate publication bias or effect modifiers for a valid inference from results. Potential effect modifiers will be the same as the subgroup analyses, i.e., for-profit bias, type of comparator, age, and sex. We will also explore these through network subgroup meta-analyses (see section below). The synthesized evidence through five steps would be useful to evidence-based decision-making in healthcare. Thus, network meta-analyses should be activated in order to guarantee the quality of health care system.

The estimation of each treatment comparison will be reported separately using the relevant effect size (RR or MD or SMD), a 95% CI, and a 95% prediction interval. Along the estimated effect sizes, we will present the ranking probabilities for each treatment being at each possible rank, as well as the surface under the cumulative ranking curve (SUCRA) or relevant *P* scores [46, 47]. A rank-heat plot will be used to depict the SUCRA values or *P* scores across all outcomes [48].

#### Individual patient data meta-analysis

Results of individual patient data meta-analysis will increase the possibility to identify subgroups of patients with specific effects of the assessed interventions. If we receive individual patient data for all eligible randomized clinical trials, we will analyze the data using a one-stage analysis model based on generalized linear mixed models. This analysis will be adjusted for the categoric baseline variables that the trials used as stratifications variables in their randomization (only the common variables that all of the trials adjust for). When analyzing continuous data, we will also adjust all analyses for the baseline value.

If we are unable to obtain sufficient individual patient data, we will secondly conduct a two-stage analysis, where at 1st stage, we will reduce available individual patient data to aggregate data for each study, and at 2nd stage, we will combine all available data in a meta-analysis.

#### Assessments of underlying statistical assumptions

We will systematically assess underlying statistical assumptions for all statistical analyses [49, 50]. In short, for all regression analyses, we will test for major interactions between each covariate and the intervention variable. We will, in turn, include each possible first order interaction between included covariates and the intervention variable. For each combination, we will test if the interaction term is significant and assess the effect size. We will only consider that there is evidence of an interaction if the interaction is statistically significant after Bonferroni adjusted thresholds (0.05 divided by number of possible interactions) and if the interaction shows a clinically significant effect. If it is concluded that the interaction is significant, we will consider both presenting an analysis separately for each (e.g., for each site if there is significant interaction between the trial intervention and “site”) and an overall analysis including the interaction term in the model [49, 50]. For detailed description of the planned assessments for underlying assumptions, please consult the recommendations of Nørskov et al. [49, 50].

### Subgroup analyses and integration of heterogeneity

#### Subgroup analyses

We will perform the following subgroup analyses when analyzing the primary outcomes (all-cause mortality and serious adverse events).

1. Trials at high risk of bias compared to trials at low risk of bias
2. Trials without for-profit bias compared to trials at unknown or known risk of for-profit bias [51]
3. Types of comparators (e.g., “active” comparator, placebo, standard care, no intervention; active placebo)
4. Age (children as defined by trialists; adults as defined by trialists; elderly as defined by trialists)
5. Sex (male/female)

We will use the formal test for subgroup interactions in Stata [21]. We will perform any unanticipated subgroup analyses, if we identify these, as more information about this virus and its treatment becomes available.

#### Sensitivity analysis

To assess the potential impact of the missing data for dichotomous outcomes, we will perform the two following sensitivity analyses on all primary and secondary dichotomous outcomes.

- “Best-worst-case” scenario: We will assume that all participants lost to follow-up in the experimental group survived, had no serious adverse events, were not admitted to intensive care, did not receive mechanical ventilation, and had no non-serious adverse events, and that all those participants lost to follow-up in the control group did not survive, had a serious adverse event, were admitted to the intensive unit, received mechanical intervention, and had a non-serious adverse event.
- “Worst-best-case” scenario: We will assume that all participants lost to follow-up in the experimental group did not survive, had a serious adverse event, were admitted to the intensive unit, received mechanical intervention, and had a non-serious adverse event, and that all those participants lost to follow-up in the control group survived, had no serious adverse events, were not admitted to intensive care, did not receive mechanical ventilation, and had no non-serious adverse events.

We will present results of both scenarios in our review. When analyzing quality of life, a “beneficial outcome” will be the group mean plus two SDs (we will secondly use one SD in another sensitivity analysis) of the group mean, and a “harmful outcome” will be the group mean minus two SDs (we will secondly use one SD in another sensitivity analysis) of the group mean [13]. To assess the potential impact of missing SDs for continuous outcomes, we will perform the following sensitivity analysis:

- Where SDs are missing and it is not possible to calculate them, we will impute SDs from trials with similar populations and low risk of bias. If we find no such trials, we will impute SDs from trials with a similar population. As the final option, we will impute the mean SD from all included trials.

We will present results of this scenario in our review. Other post hoc sensitivity analyses might be warranted if unexpected clinical or statistical heterogeneity is identified during the analysis of the review results [13].

### Summary of findings table

We will create a summary of findings table including each of the prespecified outcomes (all-cause mortality, serious adverse events, admission to intensive care, mechanical ventilation, renal replacement therapy, quality of life, and non-serious adverse events). We will use the five GRADE considerations (bias risk of the trials, consistency of effect, imprecision, indirectness, and publication bias) to assess the quality of a body of evidence [13, 52–54]. We will assess imprecision using trial sequential analysis. We will justify all decisions to downgrade the quality of evidence using footnotes, and we will make comments to aid the reader’s understanding of the review where necessary. Firstly, we will present our results in the summary of findings table based on the results from the trials with overall low risk of bias, and secondly, we will present the results based on all trials.

### Data sharing and availability

Full syntax of all statistical analyses will be published as supplementary material. All aggregate data will be published continuously. Anonymised individual patient data will also be published if possible (we will discuss this with the trialists).

### Dissemination plan

Findings of this living systematic review will be published in international peer-reviewed scientific journals. Further, a dedicated webpage for the project will be developed, where iterative versions of the living systematic review will be accommodated with visual illustrations.

## Discussion

This living systematic review with aggregate data meta-analyses, Trial Sequential Analyses, network meta-analysis, and individual patient data meta-analyses aims at comparing the effects of all interventions for treatment of COVID-19 versus placebo, standard care, no intervention, or active placebo. Primary outcomes will be all-cause mortality and serious adverse events. Secondary outcomes will be proportion of participants admitted to intensive care, proportion of participants receiving mechanical ventilation, proportion of participants receiving renal replacement therapy, quality of life, and proportion of participants with a non-serious adverse event.

This protocol has a number of strengths. The predefined methodology is based on the Cochrane Handbook for Systematic Reviews of Interventions [14], the eight-step assessment suggested by Jakobsen et al. [13], Trial Sequential Analysis [22], and GRADE assessment [52]. Hence, this protocol considers both risks of random errors and risks of systematic errors. Another strength of this protocol is that we plan to do a living systematic review, which allows us to continuously surveil the literature and update the evidence-base of existing interventions for treatment of COVID-19 resulting in a decreased timespan from evidence to clinical practice. This is particularly important in this international health care crisis. Furthermore, we plan to contact all trial authors to receive individual patient data. Often aggregate data meta-analyses and individual patient data meta-analyses tend to show similar overall results. However, an advantage of us including individual patient data meta-analyses is that it may allow us to study intervention effects in subgroups of participants.

Our protocol also has some limitations. The primary limitation is the inclusion of all types of interventions for treatment of COVID-19. This may theoretically result in a large amount of comparisons resulting in problems with multiplicity. We plan to use Trial Sequential Analysis to adjust thresholds for significance when continuously updating the review, but we do not take into account the large number of comparisons. This large risk of type 1 error will be considered when interpreting the review results.

## Supplementary information

**Additional file 1.** PRISMA-P 2015 Checklist.

**Additional file 2.** Search strategies for Covid-19.

## Data Availability

Data sharing is not applicable to this protocol article. We will publish all data including code in the supplementary material of the systematic review.

## Abbreviations

CENTRAL: Cochrane Central Register of Controlled Trials
CI: Confidence interval
CPCI-S: Conference Proceedings Citation Index—Science
CSDR: Clinical Study Data Request
EMA: European Medicines Agency
COVID-19: Corona Virus Disease 19
ECMO: Extra corporal membrane oxygenation
EMBASE: Excerpta Medica database
FDA: US Food and Drug Administration
GRADE: The Grading of Recommendations Assessment, Development and Evaluation
ICH-GCP: International Conference on Harmonization of technical requirements for registration of pharmaceuticals for human use—Good Clinical Practice
MD: Mean differences
MEDLINE: Medical Literature Analysis and Retrieval System Online
PRISMA: Preferred reporting items for systematic review and meta-analysis
PRISMA-P: Preferred reporting items for systematic review and meta-analysis—protocols
PROSPERO: International Prospective Register of Systematic Reviews
RR: Risk ratio
SARS-CoV-2: Severe acute respiratory syndrome coronavirus 2
SCI-EXPANDED: Science index citation expanded
SD: Standard deviation
SMD: Standardized mean difference
SUCCRA: Surface under the cumulative ranking curve
WHO: World Health Organization
YODA: Yale University Open Data Access
