# Supplementary material for: Interventions for treatment of COVID-19: a protocol for a living systematic review with network meta-analysis including individual patient data (The LIVING Project)
Source: Syst Rev. 2020 May 9;9:108. doi: 10.1186/s13643-020-01371-0 (PMC7210799; doi:10.1186/s13643-020-01371-0)
Supplement: Supplementary file 2 — Additional file 2. Search strategies for Covid-19. [file 13643_2020_1371_MOESM2_ESM.doc]

# Search strategies for

# Covid-19

# (S Juul)

# Preliminary searches performed 1 April 2020

**Total number of records identified: 3255 records**

**Number of duplicates excluded: 600 records**

**Number of records in final list: 2655 records**

Cochrane Central Register of Controlled Trials (CENTRAL; 2020, Issue 4) in the Cochrane Library (281 hits in CENTRAL)

#1 MeSH descriptor: [Coronavirus Infections] explode all trees

#2 ((corona adj (virus or viral or infection*)) or coronaviri* or covid* or sars-cov-2)

#3 #1 or #2

**MEDLINE Ovid (1946 to April 2020) (486 hits)**

1. exp Coronavirus Infections/

2. ((corona adj (virus or viral or infection*)) or coronaviri* or covid* or sars-cov-2).mp. [mp=title, abstract, original title, name of substance word, subject heading word, floating sub-heading word, keyword heading word, organism supplementary concept word, protocol supplementary concept word, rare disease supplementary concept word, unique identifier, synonyms]

3. 1 or 2

4. (random* or blind* or placebo*).mp. [mp=title, abstract, original title, name of substance word, subject heading word, floating sub-heading word, keyword heading word, organism supplementary concept word, protocol supplementary concept word, rare disease supplementary concept word, unique identifier, synonyms]

5. randomized controlled trial.pt.

6. controlled clinical trial.pt.

7. 4 or 5 or 6

8. 3 and 7

**Embase Ovid (1974 to April 2020) (1685 hits)**

1. exp coronaviridae/

2. ((corona adj (virus or viral or infection*)) or coronaviri* or covid* or sars-cov-2).mp. [mp=title, abstract, heading word, drug trade name, original title, device manufacturer, drug manufacturer, device trade name, keyword, floating subheading word, candidate term word]

3. 1 or 2

4. exp randomized controlled trial/

5. exp controlled clinical trial/

6. exp intermethod comparison/

7. exp double blind procedure/

8. (random* or blind* or placebo*).mp. [mp=title, abstract, heading word, drug trade name, original title, device manufacturer, drug manufacturer, device trade name, keyword, floating subheading word, candidate term word]

9. 4 or 5 or 6 or 7 or 8

10. 3 and 9

**LILACS (Bireme; 1982 to April 2020) (96 hits)**

((corona and (virus or viral or infection$)) or coronaviri$ or covid$ or sars-cov-2) [Words]

**CINAHL (Ebsco host; April 2020) (307 hits)**

S5 S3 AND S4

S4 TX (random* or blind* or placebo*)

S3 S1 OR S2

S2 TX ((corona and (virus or viral or infection*)) or coronaviri* or covid* or sars-cov-2)

S1 MH coronavirus

**BIOSIS (Web of Science; 1969 to April 2020) (269 hits)**

#3 #2 AND #1

#2 TS=(random* or blind* or placebo*)

#1 TS=((corona near (virus or viral or infection*)) or coronaviri* or covid* or sars-cov-2)

**Science Citation Index EXPANDED (1900 to April 2020) and** **Conference Proceedings Citation Index – Science (1990 to April 2020) (Web of Science) (131 hits)**

#3 #2 AND #1

#2 TS=(random* or blind* or placebo*)

#1 TS=((corona near (virus or viral or infection*)) or coronaviri* or covid* or sars-cov-2)
